# Genetic features of bovine viral diarrhea virus subgenotype 1c in newborn calves at nucleotide and synonymous codon usages

**DOI:** 10.3389/fvets.2022.984962

**Published:** 2022-08-31

**Authors:** Huihui Wang, Mengzhu Wang, Xili Feng, Yicong Li, Derong Zhang, Yan Cheng, Junlin Liu, Xiezhong Wang, Licheng Zhang, Hua La, Xiaoqian You, Zhongren Ma, Jianhua Zhou

**Affiliations:** ^1^Key Laboratory of Biotechnology and Bioengineering of State Ethnic Affairs Commission, Biomedical Research Center, Northwest Minzu University, Lanzhou, China; ^2^Gansu Tech Innovation Center of Animal Cell, Biomedical Research Center, Northwest Minzu University, Lanzhou, China; ^3^College of Life Science and Engineering, Northwest Minzu University, Lanzhou, China; ^4^Qinghai Provincial Center for Animal Disease Control and Prevention, Xining, China

**Keywords:** bovine viral diarrhea virus, newborn calves, subgenotype, synonymous codon, CpG dinucleotides

## Abstract

Bovine viral diarrhea virus (BVDV), serving as an important pathogen for newborn calves, poses threat to reproductive and economic losses in the cattle industry. To survey the infection rate and genetic diversity of BVDV in newborn calves in northern China, a total of 676 sera samples of newborn calves were collected from four provinces between 2021 and 2022. All sera samples were individually detected for BVDV infection by RT-PCR and ELISA. Our results showed that the overall serological rate was 9.76% (66/676) and the average positive rate of BVDV RNA was 8.14% (55/676) in the newborn calves. Eight BVDV strains were successfully isolated from RT-PCR positive sera samples, and four isolates displayed the cytopathic effect (CPE). Based on phylogenetic tree at the genome level, the eight strains were classified into subgenotype 1c. Moreover, the BVDV isolates had a close genetic relationship with the GSTZ strain at either nucleotide or codon usage level. Interestingly, in comparison of synonymous codon usage patterns between the BVDV isolates with CPE and ones without CPE, there were four synonymous codons (UCG, CCC, GCA, and AAC) which displayed the significant differences (*p* < 0.05) at codon usage pattern, suggesting that synonymous codon usage bias might play a role in BVDV-1c biotypes. In addition, the usage of synonymous codons containing CpG dinucleotides was suppressed by the BVDV-1c isolates, reflecting one of strategies of immune evasion of BVDV to its host. Taken together, our study provided data for monitoring and vaccination strategies of BVDV for newborn calves in northern China.

## Introduction

Bovine viral diarrhea virus (BVDV), serving as an infectious pathogen associated with reproductive, gastrointestinal, and infertility diseases of the cattle industry in the world. BVDV is an enveloped single-stranded positive sense RNA virus, and is classified into the genus *Pestivirus*, family *Flaviviridae*. In BVDV genome, the 5′ untranslated region (UTR) and 3′ UTR flank an open reading frame (ORF). Within the *Pestivirus* genus of the family *Flaviviridae*, three viral genotypes are associated with bovine viral diarrhea: BVDV-1, BVDV-2, and *Pestivirus H* (HoBi-like pestivirus, BVDV-3) (1). BVDV has an ability to cross the placenta during early pregnancy, and furthermore leads to the birth of persistently infected (PI) calves (1). Vertical transmission of BVDV happens when the virus is transmitted from the infected dam to her off-spring. During the phase of gestation, fetal infection may cause abortion, stillbirth, teratogenic effects, in addition, when the infection occurs during the first trimester, it often results in the birth of immunotolerant, persistently infected, viremic calves (2, 3). Humoral and cellular immunotolerance to BVDV strains is a unique characterization of persistent infection. Owing to the nature of persistent infection of BVDV, there is no effective treatment to fully cure an animal with BVDV infection (4). Thus, effective BVDV control programs represent that removal of PI animals (such as PI newborn calves) leads to viral extinction in the cattle herds. Generally, the genome sequences of ruminant pestiviruses change little during PI. Nevertheless, they exhibit large heterogeneity, reflecting a long history of virus-host coevolution in which avirulent strains are more successful (5).

Epidemiological analyses strongly indicate that the demographic factors such as herd size and density are regarded as important predictors for the prevalence of infection in populations where BVDV is endemic (6–8). Due to the error-prone nature of the RNA polymerases responsible for replication of viral genome, BVDV displays a high mutation for adapting to its susceptible host. BVDV-1 is the dominant genotype around the world, and at least 21 subgenotypes (BVDV-1a to BVDV-1u) have been proposed (9, 10). BVDV infection has widely spread throughout China with high prevalence and genetic diversity. With the development of the cattle industry in China, different viral variants and the corresponding high seroprevalences for BVDV infection are always observed in cattle herds in different provinces (11–16). In particular, the seroprevalence of BVDV in dairy cattle is high, approaching 57%, with a BVDV RNA positive rate of 27.1% (13). As for the circulating BVDV variants in China, there are at least 11 subgenotypes of BVDV variants infecting different domestic animals, including BVDV-1a to BVDV-1d, BVDV-1m to BVDV-1q, BVDV-1u, BVDV-2a, and BVDV-2b) (17–23). However, the previous studies were mainly conducted for adult cattle herds in China and the prevalence of BVDV infection in newborn calves appears to be ignored for investigations. Since the newborn calves with BVDV infection have a serous impair to the development of cattle industry in China, the molecular and serological studies for newborn calves with BVDV infection can display valuable information about the BVDV strains circulating in a population and thereby conduct an effective control program, guide vaccination strategy and trace likely infection sources. In this study, we conducted a survey on evaluating the prevalence of BVDV infection and genetic diversity of BVDV isolates in newborn calves in northern China.

## Materials and methods

### Blood sample collection for newborn calves

This study was approved by the Animal Ethics Committee of College of Life Science and Engineering, Northwest Minzu University (Approval No. CLSEAEC 2020-003). The newborn calves within 14 birth days were handled in accordance with good animal practices required by the Animal Ethics Procedures and Guidelines of the People's Republic of China. From the four provinces (Ningxia, Shanxi, Shandong, and Inner Mongolia) in China, the newborn calves were selected in random, after obtaining the verbal consent from the owners of cattle farm. Finally, a total of 676 blood samples were collected, and extracting serum was conducted by the conventional operation (24). The sera samples were kept at −20°C until further analysis.

### Serological detection for BVDV infection

All sera samples for total antibodies against BVDV were tested by using a competition ELISA test kit (Ingenasa, Spain). As for the competition ELISA kit, the recombinant peptide of p80/p125 antigen from pestivirus is used for the viral antigen.

### RT-PCR detection and virus isolation

Total RNA was extracted from each serum sample using TIANampvirus DNA/RNA kit (TIANGEN Biotech, China). The extracted RNA was reverse-transcribed using the M-MLV Reverse transcriptase kit (TaKaRa, China) following the manufacturer. Twelve primer sets were designed to amplify overlapping regions covering the whole BVDV genome (Supplementary Table 1). The primer set for amplifying 5'UTR sequence was used for preliminarily detecting BVDV RNA. After gel purification, the amplification product was directly sequenced by Tsingke Biotechnology Co., Ltd.

The positive serum sample, which contained BVDV RNA, was inoculated onto MDBK monolayer cultures, 25 cm flasks with a 1 mL inoculum (1:10 dilution of original samples) and the total volume was 5 mL. The cell cultures were frozen and thawed three times and passaged ten times at 5 days interval. The processed MDBK culture needed to be observed to identify absence or presence of the cytopathic effect (CPE). Furthermore, for estimating viral titer of BVDV strain with CPE, the 50% tissue culture infectivity dose (TCID_50_) method was used for the corresponding BVDV titer assessment (25, 26).

### Analyses for genetic features of BVDV isolates

#### Phylogenetic analysis

For clarifying genetic relationship between the filed BVDV isolates and BVDV strains circulating in China, 28 strains with the whole viral genome recorded in GenBank were used in this study (Supplementary Table 2). The phylogenetic reconstruction for genetic genotyping of the BVDV isolates were compiled. The phylogenetic tree was inferred by viral genome by producing Maximum Likelihood (ML) trees with Tamura-Nei model plus Gamma distribution of base substitution using MAGE 7.0 software. Here, we selected the GTR+G+I model for performing ML tree to display genetic diversity of the target BVDV strains, according to the minimum of Bayesian information criterion (BIC) which corresponds to the maximum likelihood fits of the specific nucleotide substitution model.

#### Analyzing synonymous codon usage patterns for the BVDV isolates

The relative synonymous codon usage value (RSCU) for a synonymous codon is the ratio of its observed frequency to its expected frequency without biased usage (the mean frequency of all synonymous members encoding their specific amino acid) (27). RSCU would be an objective representation of the synonymous codon usage bias without confounding the influence of amino acid composition for different BVDV strains. In general, when the RSCU value is higher than 1.0, the corresponding synonymous codon displays a positive codon usage trend. In contrast, when the RSCU value is <1.0, the corresponding synonymous codon reflects a negative codon usage trend (28, 29). To identify the extent of 59 synonymous codon usage bias, RSCU values of higher than 1.6 and <0.6 were defined as “overrepresented” and “underrepresented” codons, respectively (30).

In addition, principal component analysis (PCA) is a multivariate statistical method which reduces data dimensionality by performing a covariance analysis for a data matrix (31). As for genetic diversity of the BVDV isolates in respect with the overall codon usage, the PCA analysis was performed for RSCU data of viral ORF. The plot which displayed genetic diversity of these strains was composed of the first variant (*f*_1_' data) and the second variant (*f*_2_' data), revealing the overall codon usage pattern derived from the 59 synonymous codon usage variants for each strain in this study.

### Statistical analysis

One-way ANOVA method was used to compare the means of two or more groups containing numerical response data using the software SPSS 16.0 for Windows, and significant difference can be identified when *p* value was < 0.05. Linear regression was used for modeling the relationship between a scalar dependent variable and one independent variable using the software GraphPad Prism 6 for Windows.

## Results

### Positive results represented by ELISA and RT-PCR for newborn calves with BVDV infection

To estimate the serological prevalence of newborn calves with BVDV infection, each serum sample was tested for the anti-BVDV antibodies. The overall serological rate was 9.76% (66/676) for newborn calves in this study. It ranges from 4 to 16% across northern China (Table 1). Among the four provinces, the serological rate for BVDV infection was highest in Shandong province, followed by Inner Mongolia and Ningxia, while that of Shanxi province was lowest (Table 1). Turning to RT-PCR test for BVDV RNA, 55 positive samples were found from all sera samples and the positive rate is 8.14%. In details, the positive rate (12.77%) was highest in Ningxia, followed by 4% in Shanxi and 2% in Shandong province, whereas the positive rate (1%) in Inner Mongolia was lowest (Table 1). Moreover, 4 sera samples, which contained antibodies for BVDV and BVDV RNA, were identified in the 376 sera samples from Ningxia province (Table 1).

**Table 1:** The information about the target serum sample which contain BVDV genome and antibodies for BVDV.

**Province**	**Total samples**	**^a^The number (%) of serum sample with antibodies for BVDV**	**^b^The number (%) of serum sample with BVDV RNA**	**^c^The number of serum sample with antibodies for BVDV and BVDV RNA**
Ningxia	376	34 (9.04%)	48 (12.77%)	4 (1.06%)
Inner Mongolia	100	12 (12%)	1 (1%)	None
Shandong	100	16 (16%)	2 (2%)	None
Shanxi	100	4 (4%)	4 (4%)	None

^a^Means the positive results in ELISA assay for antibodies for BVDV in sera samples derived from the specific province.

^b^Means the positive results in RT-PCR test for BVDV genome in sera samples derived from the specific province.

^c^Means the positive results in RT-PCR test for BVDV genome and ELISA assay for antibodies for BVDV in sera samples at the same time.

### Eight BVDV strains isolated from positive sera samples

Each serum sample containing BVDV RNA was used for virus isolation. From the 55 positive sera samples, eight BVDV strains were successfully isolated. According to biotype assay, there were four BVDV strains with CPE (21NX-300, 21NX-53, 21SD-16, and 22NX-9) and four strains without CPE (21NX-69, 21NM-44, 21SD-5, and 22NX-197) (Table 2). To estimate viral titer of the four BVDV strains with CPE, the TCID_50_ assay showed the different titers ranging from 10^5.0^ to 10^7.0^.

**Table 2:** The information about the eight filed BVDV isolates from sera samples.

**Strain**	**Biotype**	**^c^TCID_50_/mL**
21NX-53	^a^CP	10^7.0^
21NX-300	CP	10^6.4^
21SD-16	CP	10^6.2^
22NX-9	CP	10^5.0^
21NX-69	^b^NCP	Not application
21NM-44	NCP	Not application
21SD-5	NCP	Not application
22NX-197	NCP	Not application

^a^ “CP” means cytopathogenicity.

^b^“NCP” means non-cytopathogenicity.

^c^The values of TCID_50_/mL were derived from BVDV which were processed by 4 passages in MDBK cells.

### The eight BVDV strains being subgenotype 1c

**Figure 1:**
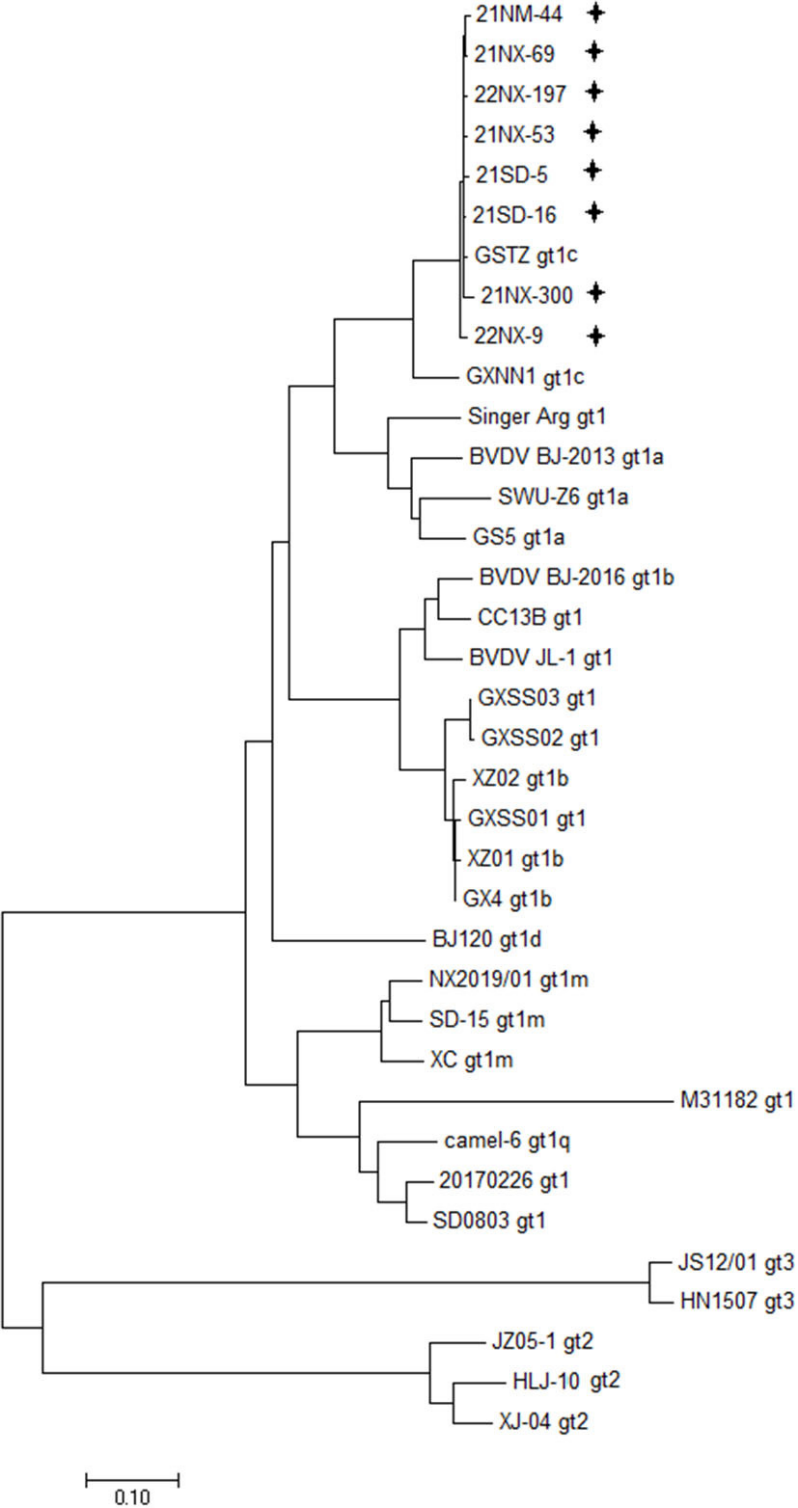
Phylogenetic analysis based bon the whole genome. A phylogenetic tree was created using the whole genomes of eight BVDV isolates and 28 reference strains retrieved from the GenBank database. “**+**” BVDV isolates identified in the four provinces in this study. All isolates were clustered in BVDV-1c.

Depending on the specific PCR primers, the eight BVDV strains were sequenced for the whole genome. The 12 overlapping fragments, ranging from 931 to 1,168 nt in size, were obtained (Supplementary Table 1). The eight viral genomes ranged from 12,132 to 12,453 in length, and their ORF ranged from 11,691 to 11,727 nt in length, encoding a polyprotein ranging from 3,896 amino acids to 3,989 amino acids in length (Supplementary Table 3). Moreover, the 5'UTR and 3'UTR of these BVDV isolates ranged from 282 to 317 nt and ranged from 153 to 409 nt, respectively. Furthermore, 28 BVDV strains reported in China, which were sequenced for the whole genome available in the GenBank database, were used for clarifying genetic diversity for the eight BVDV strains at the genome level (Supplementary Table 2). A phylogenetic tree including representations of BVDV-1 with six subgenotypes (1a-1d, 1m, and 1q), BVDV-2 and BVDV-3 displayed that the eight BVDV strains were clustered in the same terminal node with the two strains of subgenotype 1c (GSTZ strain, GenBank accession no. MF172980.1 and GXNN1 strain, GenBank accession no. MT079816.1) (Figure 1). Despite the strains GSTZ and GXNN1 belonging to BVDV-1c, these BVDV strains had the closer genetic relationships with the strain GSTZ than GXNN1. Since the strain GSTZ was isolated from Gansu province in northern China and the strain GXNN1 was isolated from Guangxi province in southern China, geographic factor appeared to participate in evolutionary pathway of the eight BVDV strains to some degree.

### Strong selective forces suppressing codon usage for the BVDV isolates

According to nucleotide usages of the eight strains (Supplementary Table 4), the nucleotide A content was highest (average value = 31.96 ± 0.05%), followed by those of nucleotide G (26.09 ± 0.05%) and U (22.19 ± 0.04%), while the nucleotide C content was lowest (19.76 ± 0.04%). Moreover, the nucleotides A (average value = 30.52 ± 0.08%) and G (26.81 ± 0.11%) at the third codon position were similar with the corresponding nucleotide content at codon level, while the nucleotide U content (20.22 ± 0.11%) was less than that of nucleotide C (22.44 ± 0.12%) at the third codon position (Supplementary Table 4). For investigating the role of nucleotide composition constraint in synonymous codon usage bias, the RSCU analysis was performed for the eight BVDV strains. Generally, the eight BVDV strains displayed the similar synonymous codon usage patterns. All overrepresented synonymous codons did not follow the codon usage pattern with G/C end or A/U end, and all synonymous codons containing CpG dinucleotides were slightly selected by the eight BVDV strains (Supplementary Table 5). Although the nucleotide composition constraint of BVDV ORF had a strong effect on the usage of overrepresented codons including AUA (Ile), UCA (Ser), CCA (Pro), AGA, and AGG (Arg) and the underrepresented ones (CUU, CUC for Leu, GUU for Val, and CGU for Arg), the usage patterns for some underrepresented ones appeared to be little influenced, including UCG for Ser, CCG for Pro, ACG for Thr, GCG for Ala, CGA, and CGG for Arg (Supplementary Table 5). The genetic features reflected the strong selective forces suppressing the synonymous codon usages for the eight BVDV strains. Interestingly, the comparisons of synonymous codon usage patterns between the BVDV isolates with CPE and ones without CPE showed the significantly different usage patterns for UCG encoding Ser (*p* = 0.017), CCC encoding Pro (*p* = 0.004), GCA encoding Ala (*p* = 0.03) and AAC encoding Asn (*p* = 0.042).

**Figure 2:**
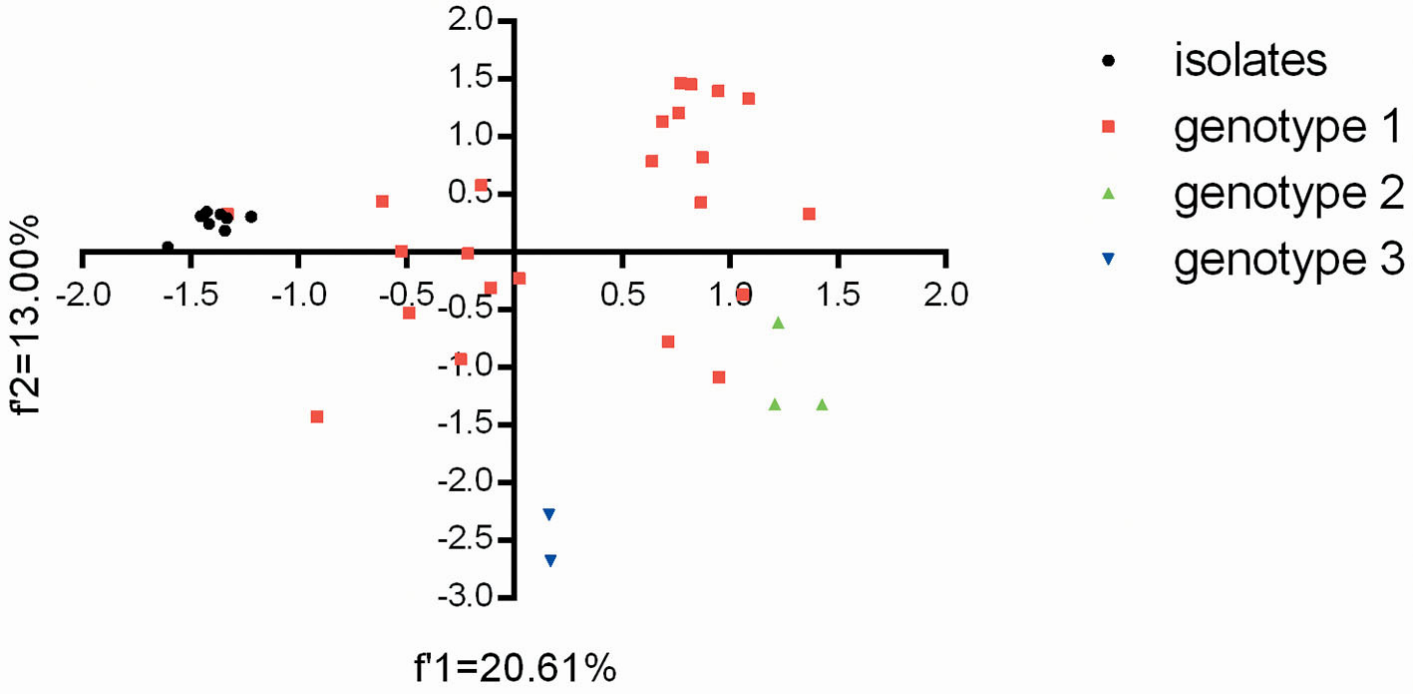
The plot for the overall codon usage is visualized by the PCA method for these BVDV strains in this study.

Based on the overall codon usage pattern (RSCU data) of the BVDV isolates (Supplementary Table 5), PCA was performed for genetic relationship between the eight BVDV strains and other strains reported in China. For the relevant 59 multi-dimensional datasets, PCA reduces the dimensionality of the data so that efficient visualization that captures most of the variation can take place. Here, the first two axes from the PCA analysis were used to establish a 2D visualization of genetic diversity for different BVDV strains in China. After PCA analysis, the projection of BVDV strains displayed that the eight BVDV isolates had the close genetic relationships with the strain GSTZ and showed obviously differences from others (Figure 2). Moreover, the two BVDV-3 strains had an obviously difference from others and exhibited the genotype-specific codon usage, but the three BVDV-2 strains appeared to display a closer genetic relationship with the three BVDV-1 strains (“20170226,” “camel-6,” and “SD0803”) than other BVDV-1 strains (Figure 2). The result reflected that BVDV-3 strains, which emerged in China, owned a significant difference from those of BVDV-1 and BVDV-2 at codon usage, and the BVDV-1 and BVDV-2 strains did not display the genotype-specific codon usage. In addition, the overall codon usage patterns for these BVDV-1 strains obviously exhibited a high synonymous codon usage variant. This result was consistent with the previous reports for BVDV-1 strains with high mutation variations (9, 32, 33).

## Discussion

BVDV is the most prevalent in cattle industry in China. The previous study showed that the prevalence of BVDV RNA was 22.64% in cattle herds in China (10). A systematic review pointed out that BVDV RNA prevalence was 27.1% in dairy cattle in China (13). However, relatively few studies have been conducted for BVDV RNA detection, viral seroprevalence, virus isolation, genotyping, and its distribution in newborn calves. Vertical transmission of BVDV occurs when the virus is transmitted from the infected dam to her off-spring. Fetal infection may, dependent on the phase of gestation, result in abortion, stillbirth, teratogenic effects, or, when the infection occurs during the first trimester, in the birth of immunotolerant, persistently infected, viremic calves (2, 3). Our study has extended the previous investigation and displayed the serological and RNA prevalence for BVDV infection in newborn calves. Furthermore, our study demonstrated that the overall positive rate of BVDV RNA was 8.14% (55/676) and serological prevalence was 9.76% (66/676) in newborn calves in northern China. Currently, there are three main detection methods (RT-PCR, ELISA, and virus isolation) for BVDV infection in cattle herds. To some degree, limitations in the samplings and detection methods might influence the validity of BVDV infection in newborn calves. In general, the prevalence of BVDV infection in newborn calves in northern China was significant weaker than the overall BVDV infection in cattle population, suggesting that BVDV infection in newborn calves via vertical transmission might not serve as an important factor of BVDV epidemic in cattle herds. Since newborn calves with BVDV infection, especially PI newborn calves, pose threat to cattle industry, these calves which shed enormous amounts of BVDV throughout their lives are the major source for the spread and perpetuation of the virus within individual cattle herds and for the transmission to previously not affected holdings (6, 34, 35). Thus, newborn calves with BVDV infection should be seriously considered an important target of BVDV eradication via rapid detection or vaccination strategy.

Compared with RT-PCR for BVDV RNA and ELISA for BVDV antigens / antibodies, virus isolation is the standard method of detection of BVDV-infected newborn calves. The BVDV infected cattle, especially PI cattle, can secrete large amounts of BVDV in their serum (36, 37). Hence, the serum from the newborn calf is a valid sample for BVDV isolation. In our study, eight BVDV strains (four CP strains and four NCP ones) were successfully isolated from clinical serum of newborn calves. These BVDV isolates with different biotypes further provide more biological information. After the phylogenetic genotyping analysis, the eight BVDV isolates were classified into subgenotype 1c and displayed a close genetic relationship with the strain GSTZ which was isolated from yak in Gansu province (Figure 1). Previous study described the circulation of at least six subgenotypes of BVDV-1 in China (1a, 1b, 1c, 1d 1m, and 1q) with predominance of BVDV-1a and BVDV-1d (32, 37, 38). Currently, BVDV-1a and BVDV-1b are still the most often detected subgenotypes in the big region including Asia, Europe and Americas (39). In addition, although BVDV-2 and BVDV-3 strains have been isolated in China (16, 19, 40, 41), our study showed that the two BVDV species do not seem to circulate in newborn calves in northern China. BVDV-1c may be unique for newborn calves in northern China and needs to be monitored from now on. The previous study pointed out that BVDV-1a and BVDV-1c were mainly circulating in cattle population (yak, dairy cattle, and beef cattle) in China (37). In fact, BVDV-1c is by far the most commonly circulating in Australia, and BVDV-1c infected cattle with no overt respiratory signs commonly displayed transient pyrexia and leukopenia (42). In our study, BVDV-1c plays important roles in BVDV infection for newborn calves. The BVDV-1c infection in newborn calves might provide a potential pathway for circulating BVDV-1c strains in cattle population.

Since BVDV is an RNA virus with high mutation rate, analyses for the frequency and number of subgenotypes of BVDV in newborn calves are helpful to clarify viral evolutionary trend and the potential infectious sources. We firstly showed the genetic features of the BVDV isolates in newborn calves at both nucleotide and synonymous codon usage levels. Apart from the genetic diversity of the eight BVDV strains (Figure 1), the BVDV isolates had similar codon usage patterns with strain GSTZ and an obvious evolutional boundary separating from other BVDV-1 subgenotypes, BVDV-2 and BVDV-3 strains circulating in China (Figure 2) further suggesting that evolutionary pathway of BVDV-1c regardless of strains may be unique in comparison with other subgenotypes in China. The isolated BVDV-1c strains with heterogeneity of codon usage could raise concern related to the emergence and spread of new BVDV variants in newborn calves, with possible implications for animal health and disease control. According to nucleotide and synonymous codon usage patterns (Supplementary Tables 4, 5), nucleotide composition constraint and natural selective forces cooperated for driving synonymous codon usage bias of the eight BVDV strains. Moreover, in comparison of each synonymous codon usage bias between BVDV isolates with CP biotype and ones with NCP biotype, it was interestingly found that four synonymous codons (UCG, CCC, GCA, and AAC) displayed the significant differences at usage patterns. This genetic feature suggests that synonymous codon usage bias of BVDV might participate in shaping BVDV biotype. In general, synonymous codon usage bias is able to participate in most aspects of viral life cycle. This biased synonymous codon usage is not neutral but involved in nucleotide usage bias (28, 29, 43), mRNA stability (44, 45), translation accuracy and efficiency (46, 47), and protein folding formation (48). Of note, BVDV did not follow nucleotide usage pattern of its susceptible host (i.e., newborn calf) in which the genome of cells contains high GC content to perform viral life cycle, but it accumulated adenine content in its ORF (Supplementary Table 4) and suppressed the selection of synonymous codons with CG dinucleotides (Supplementary Table 5). It can be explained that BVDV ORF tends to suppress usages of synonymous codons containing CpG dinucleotides in order to weaken immune responses from the infected host. This genetic phenomenon can be explained by the observations that natural selection derived from host-dependent selection plays an important role in eliminating CG dinucleotides in the viral genome (49, 50).

## Conclusion

The distribution of subgenotypes in newborn calves in northern China exhibits a difference from other regions in China. Despite BVDV-1a, 1b, and 1c mainly circulating in China, these BVDV-1c appears to be main infectious source for newborn calves in northern China, and the isolated BVDV-1c strains display a unique evolutionary trend from other BVDV strains isolated from China at either nucleotide or codon usage pattern. Further studies are required to fully understand the variability and importance of the BVDV-1c. Monitoring of these BVDV isolates circulating in newborn calves is a useful indicator with respect of designing an effective vaccination program or a reliable diagnostic detection.

## Data availability statement

The datasets presented in this study can be found in online repositories. The names of the repository/repositories and accession number(s) can be found in the article/Supplementary materials.

## Author contributions

HW, JZ, and ZM: conceptualization. HW, MW, XF, XW, LZ, HL, XY, YL, and DZ: methodology. YC and JL: software. HW, DZ, MW, XF, YL, and YC: formal analyses. JZ and ZM: writing—review and editing. XW, LZ, HL, and XY: sera samples collecting. All authors contributed to the article and approved the submitted version.

## Funding

The work was supported by the Fundamental Research Funds for the Central Universities (No. 31920220069) and the Fundamental Research Funds for Introduction of Talent Research Fund of Northwest Minzu University (No. xbmuyjrc202225).

## Conflict of interest

The authors declare that the research was conducted in the absence of any commercial or financial relationships that could be construed as a potential conflict of interest.

## Publisher's note

All claims expressed in this article are solely those of the authors and do not necessarily represent those of their affiliated organizations, or those of the publisher, the editors and the reviewers. Any product that may be evaluated in this article, or claim that may be made by its manufacturer, is not guaranteed or endorsed by the publisher.
