# Supplementary material for: Genetic features of bovine viral diarrhea virus subgenotype 1c in newborn calves at nucleotide and synonymous codon usages
Source: Front Vet Sci. 2022 Aug 31;9:984962. doi: 10.3389/fvets.2022.984962 (PMC9470862; doi:10.3389/fvets.2022.984962)
Supplement: Supplementary file 1 [file Data_Sheet_1.doc]

**Genetic features of bovine viral diarrhea virus subgenotype 1c in newborn calves at nucleotide and synonymous codon usages**

Huihui Wang^1,2,3,#^, Mengzhu Wang^1,2,3,#^, Xili Feng^1,2,3^, Yicong Li^1,2,3^, Derong Zhang^1,2^, Yan Cheng^1,2,3^, Junlin Liu^3^, Xiezhong Wang^4^, Licheng Zhang^4^, Hua La^4^, Xiaoqian You^4^, Zhongren Ma^1,2^, Jian-hua Zhou^1,2^,*

1.Key Laboratory of Biotechnology and Bioengineering of State Ethnic Affairs Commission, Biomedical Research Center, Northwest Minzu University, Lanzhou 730030

2. Gansu Tech Innovation Center of Animal Cell, Biomedical Research Center, Northwest Minzu University Lanzhou 730030

3. College of Life Science and Engineering, Northwest Minzu University, Lanzhou, Gansu，730010, China

4. Qinghai Provincial Center for Animal Disease Control and Prevention, Xining, China

^#^The two authors have an equal contribution to this work

*****Corresponding author: zhoujianhua@xbmu.edu.cn

**
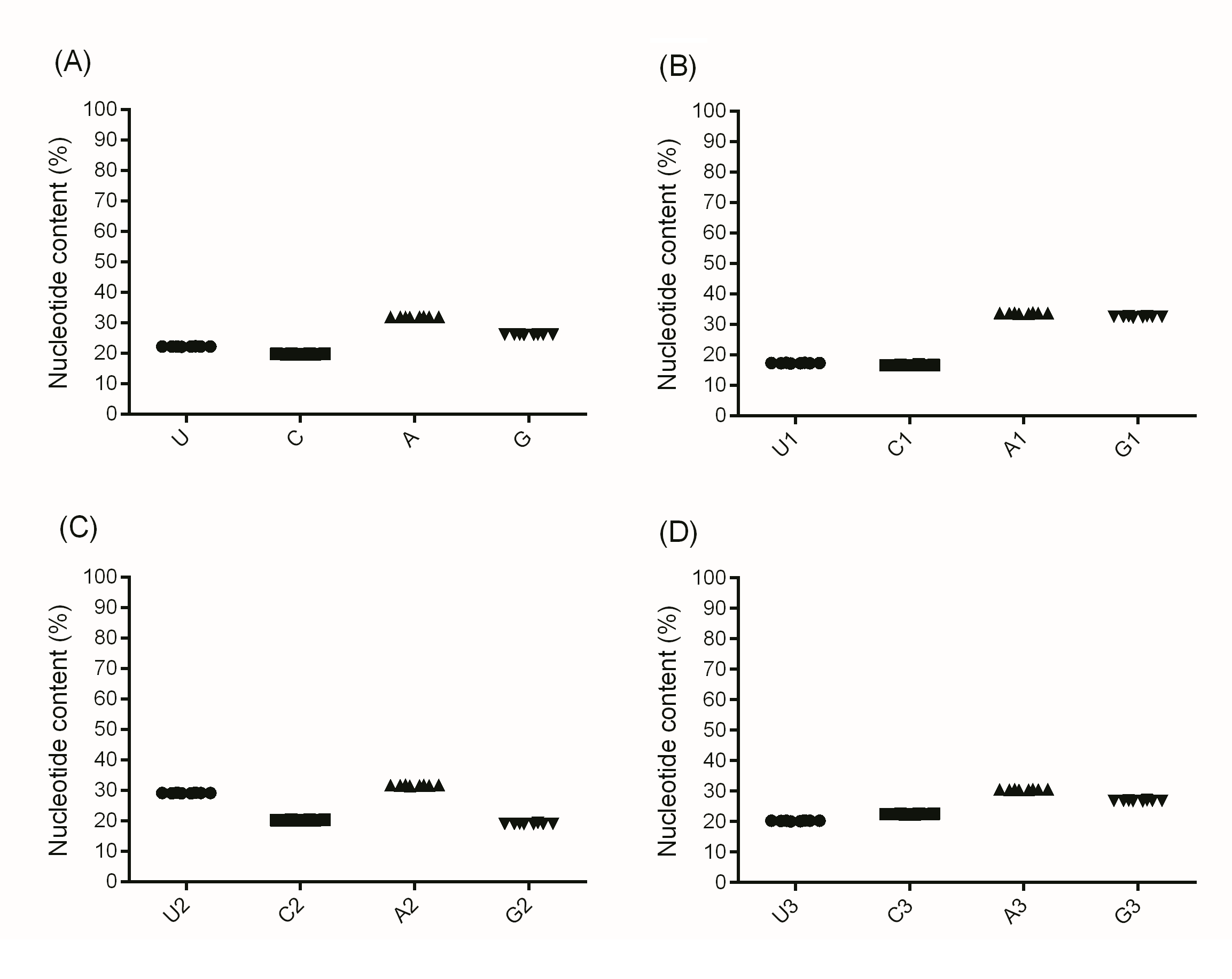
**

**Figure S1** Nucleotide contents (%) at the gene level of the eight BVDV isolates. (A) The overall nucleotide content (%) for each base; (B) The nucleotide content (%) for each base at the first codon position; (C) The nucleotide content (%) for each base at the second codon position; (D) The nucleotide content (%) for each base at the third codon position

**Table S1 The infor**mation about primers for amplifying the whole genome of the field BVDV isolates

| Primers | Primer Sequences (5’-3’) | Product lengths (bp) |
| --- | --- | --- |
| BVDV1F | TGAATCCCCTTCAGCGAAGGC | 1038 |
| BVDV1R | GTTGTGGTACAAACCATCCTG |
| BVDV2F | AGGATAGCAGGACTAAACCAC | 1016 |
| BVDV2R | GTCAAACATCCCGGGGCCTATG |
| BVDV3F | GCCTGCTTGCCCAAGAACAC | 1050 |
| BVDV3R | GTCCTCCCCCACAATTTTCTG |
| BVDV4F | TCACAGACAAGGCTGTATTAC | 1022 |
| BVDV4R | CAGCTATATCCACTCCTGGC |
| BVDV5F | CTGAGGGTCACTGGGCTAAC | 1112 |
| BVDV5R | GCGGTGTATTGTATGAAACC |
| BVDV6F | AGGGAAGGTAACTTTGAAGGC | 1036 |
| BVDV6R | CTTCTTTCAAATCCCCTATTC |
| BVDV7F | GCAGAGTCAGTCTACCAATATATG | 1003 |
| BVDV7R | CAGTTCTGGCCATGATGTTC |
| BVDV8F | CTAAACAACCTGCTCATCTC | 1021 |
| BVDV8R | CTGGTTCCACCACCTTTGAAAG |
| BVDV9F | CGCATTGGCAACCTATACATAC | 1168 |
| BVDV9R | CTTAGTTATGGTCGCACAGTC |
| BVDV10F | GATACACTGGGGTCGGATTC | 1146 |
| BVDV10R | GTTGGTAAGCTGATGCCATG |
| BVDV11F | CAACTGGCACAAGGTAACTG | 1351 |
| BVDV11R | ATCTGTAGGCAACTTTCATC |
| BVDV12F | GAGAGGATGAAAGTTGCCTAC | 931 |
| BVDV12R | GAGGTCTTTTCCTAGTCCAAC |

Table S2 The information about BVDV strains with the whole genome emerging in China

| GenBank accession number | Strain | Time | ^*^Gt | Host |
| --- | --- | --- | --- | --- |
| KF501393.1 | BVDV JL-1 | 2009 | 1 | Cattle |
| MH490943.1 | BVDV BJ-2016 | 2016 | 1b | Cattle |
| MH490942.1 | BVDV BJ-2013 | 2013 | 1a | Cattle |
| MF693403.1 | SWU-Z6 | 2016 | 1a | Yak |
| MH166806.1 | XC | 2015 | 1m | Cattle |
| MK102095.1 | 20170226 | 2017 | 1 | Cattle |
| KC695810.1 | camel-6 | 2010 | 1q | Camelus bactrianus |
| MW014288.1 | GXSS03 | 2018 | 1 | Cattle |
| MW014287.1 | GXSS02 | 2018 | 1 | Cattle |
| MW014286.1 | GXSS01 | 2018 | 1 | Cattle |
| MN623291.1 | NX2019/01 | 2019 | 1m | Cattle |
| MH133206.1 | Singer Arg | ------- | 1 | Cattle |
| MF172980.1 | GSTZ | 2012 | 1c | Yak |
| MF278652.1 | XZ02 | 2016 | 1b | Cattle |
| MF278651.1 | XZ01 | 2016 | 1 | Cattle |
| KT943518.1 | BJ120 | 2012 | 1d | Cattle |
| KR866116.1 | SD-15 | 2015 | 1m | Cattle |
| JN400273.1 | SD0803 | 2008 | 1 | Cattle |
| KJ689448.1 | GX4 | 2012 | 1b | Cattle |
| KJ541471.1 | GS5 | 2013 | 1a | Cattle |
| JQ799141.1 | M31182 | 2010 | 1 | Yak |
| KF772785.1 | CC13B | 2013 | 1 | Cattle |
| MT079816.1 | GXNN1 | 2018 | 1c | Cattle |
| FJ527854.1 | XJ-04 | 2004 | 2 | Cattle |
| GQ888686 | JZ05-1 | 2005 | 2 | Cattle |
| JF714967.1 | HLJ-10 | 2011 | 2 | Cattle |
| KU563155.1 | HN1507 | 2015 | 3 | Capra hircus |
| JX469119.1 | JS12/01 | 2012 | 3 | Cattle |

^*^Gt means the word ‘genotype’

Table S3 The information about the complete genomes of isolated BVDV strains

| Strain | GenBank accession no. | Genome (nt) | 5’UTR/3’UTR  (nt) | ORF (nt) | Polyportein (aa) |
| --- | --- | --- | --- | --- | --- |
| 21NX-53 | ON411192 | 12132 | 282 / 153 | 11697 | 3898 |
| 21NX-300 | ON098979 | 12149 | 299 / 159 | 11691 | 3896 |
| 21SD-16 | ON624287 | 12238 | 315 / 217 | 11706 | 3901 |
| 22NX-9 | ON624285 | 12453 | 317 / 409 | 11727 | 3908 |
| 21NX-69 | ON165517 | 12198 | 282 / 219 | 11697 | 3898 |
| 21NM-44 | ON411191 | 12191 | 283 / 208 | 11700 | 3989 |
| 21SD-5 | ON165518 | 12216 | 283 / 230 | 11703 | 3900 |
| 22NX-197 | ON411193 | 12202 | 282 / 217 | 11703 | 3900 |

Table S4 The nucleotide usage patterns of ORF for the eight BVDV isolates

|  | U (%) | C (%) | A (%) | G (%) | U3 (%) | C3 (%) | A3 (%) | G3 (%) |
| --- | --- | --- | --- | --- | --- | --- | --- | --- |
| 21NM-44 | 22.26 | 19.74 | 32.03 | 25.97 | 20.36 | 22.26 | 30.59 | 26.79 |
| 21NX-53 | 22.19 | 19.79 | 31.94 | 26.08 | 20.13 | 22.60 | 30.44 | 26.83 |
| 21NX-69 | 22.12 | 19.77 | 31.98 | 26.13 | 20.01 | 22.54 | 30.39 | 27.06 |
| 21NX-300 | 22.20 | 19.82 | 31.88 | 26.11 | 20.30 | 22.30 | 30.56 | 26.84 |
| 21SD-5 | 22.21 | 19.72 | 31.98 | 26.09 | 20.28 | 22.48 | 30.45 | 26.79 |
| 21SD-16 | 22.18 | 19.79 | 31.93 | 26.10 | 20.19 | 22.42 | 30.60 | 26.78 |
| 22NX-9 | 22.23 | 19.72 | 31.92 | 26.14 | 20.26 | 22.44 | 30.62 | 26.68 |
| 22NX-197 | 22.18 | 19.73 | 31.97 | 26.11 | 20.25 | 22.51 | 30.50 | 26.74 |

Table S5 RSCU data for the eight BVDV strains

|  | 21NX-53 | 21NX-300 | 21SD-16 | 22NX-9 | 21NM-44 | 21NX-69 | 21SD-5 | 22NX-197 |
| --- | --- | --- | --- | --- | --- | --- | --- | --- |
| UUU(F) | 0.97 | 0.94 | 0.97 | 0.99 | 0.97 | 0.97 | 0.97 | 0.97 |
| UUC(F) | 1.03 | 1.06 | 1.03 | 1.01 | 1.03 | 1.03 | 1.03 | 1.03 |
| UUA(L) | 0.89 | 0.89 | 0.90 | 0.94 | 0.91 | 0.89 | 0.86 | 0.87 |
| UUG(L) | 1.36 | 1.30 | 1.35 | 1.32 | 1.36 | 1.39 | 1.38 | 1.35 |
| CUU(L) | 0.50 | 0.53 | 0.52 | 0.50 | 0.50 | 0.50 | 0.51 | 0.51 |
| CUC(L) | 0.52 | 0.52 | 0.49 | 0.50 | 0.50 | 0.50 | 0.51 | 0.51 |
| CUA(L) | 1.33 | 1.35 | 1.34 | 1.32 | 1.35 | 1.31 | 1.34 | 1.35 |
| CUG(L) | 1.40 | 1.41 | 1.41 | 1.41 | 1.38 | 1.41 | 1.41 | 1.42 |
| AUU(I) | 0.61 | 0.63 | 0.62 | 0.62 | 0.65 | 0.61 | 0.62 | 0.62 |
| AUC(I) | 0.71 | 0.69 | 0.70 | 0.71 | 0.71 | 0.70 | 0.70 | 0.70 |
| AUA(I) | **1.68** | **1.68** | **1.68** | **1.66** | **1.65** | **1.69** | **1.68** | **1.69** |
| GUU(V) | 0.56 | 0.57 | 0.57 | 0.58 | 0.58 | 0.58 | 0.57 | 0.57 |
| GUC(V) | 1.03 | 0.99 | 1.01 | 1.02 | 0.99 | 1.00 | 1.00 | 1.03 |
| GUA(V) | 0.96 | 0.96 | 0.96 | 0.96 | 0.98 | 0.97 | 1.00 | 0.95 |
| GUG(V) | 1.45 | 1.48 | 1.46 | 1.44 | 1.45 | 1.45 | 1.42 | 1.45 |
| UCU(S) | 0.74 | 0.75 | 0.77 | 0.71 | 0.76 | 0.74 | 0.78 | 0.73 |
| UCC(S) | 0.77 | 0.72 | 0.74 | 0.71 | 0.74 | 0.74 | 0.75 | 0.70 |
| UCA(S) | **1.80** | **1.73** | **1.74** | **1.80** | **1.74** | **1.74** | **1.70** | **1.76** |
| *UCG(S) | 0.09 | 0.12 | 0.09 | 0.09 | 0.12 | 0.15 | 0.12 | 0.15 |
| AGU(S) | 1.30 | 1.28 | 1.27 | 1.30 | 1.26 | 1.26 | 1.30 | 1.26 |
| AGC(S) | 1.30 | 1.40 | 1.39 | 1.39 | 1.38 | 1.38 | 1.36 | 1.40 |
| CCU(P) | 0.98 | 1.03 | 0.98 | 1.04 | 1.05 | 1.01 | 1.01 | 1.05 |
| *CCC(P) | 0.95 | 0.94 | 0.96 | 0.94 | 0.88 | 0.92 | 0.91 | 0.90 |
| CCA(P) | **1.67** | **1.61** | **1.66** | **1.62** | **1.63** | **1.66** | **1.67** | **1.63** |
| CCG(P) | 0.40 | 0.41 | 0.40 | 0.40 | 0.44 | 0.41 | 0.42 | 0.41 |
| ACU(T) | 0.95 | 0.91 | 0.91 | 0.92 | 0.92 | 0.89 | 0.90 | 0.91 |
| ACC(T) | 1.02 | 1.05 | 1.04 | 1.01 | 1.03 | 1.05 | 1.05 | 1.04 |
| ACA(T) | 1.58 | 1.58 | **1.61** | **1.61** | 1.60 | 1.59 | 1.60 | **1.61** |
| ACG(T) | 0.45 | 0.46 | 0.45 | 0.46 | 0.45 | 0.48 | 0.45 | 0.43 |
| GCU(A) | 0.90 | 0.96 | 0.90 | 0.90 | 0.92 | 0.88 | 0.90 | 0.91 |
| GCC(A) | 1.26 | 1.20 | 1.28 | 1.29 | 1.25 | 1.34 | 1.25 | 1.27 |
| *GCA(A) | 1.52 | 1.55 | 1.52 | 1.52 | 1.48 | 1.49 | 1.51 | 1.51 |
| GCG(A) | 0.31 | 0.30 | 0.31 | 0.30 | 0.35 | 0.29 | 0.35 | 0.31 |
| UAU(Y) | 0.91 | 0.92 | 0.94 | 0.98 | 0.93 | 0.94 | 0.92 | 0.94 |
| UAC(Y) | 1.09 | 1.08 | 1.06 | 1.02 | 1.07 | 1.06 | 1.08 | 1.06 |
| CAU(H) | 0.72 | 0.78 | 0.71 | 0.65 | 0.72 | 0.68 | 0.72 | 0.72 |
| CAC(H) | 1.28 | 1.22 | 1.29 | 1.35 | 1.28 | 1.32 | 1.28 | 1.28 |
| CAA(Q) | 0.98 | 1.01 | 0.97 | 0.94 | 1.01 | 0.98 | 0.97 | 0.97 |
| CAG(Q) | 1.02 | 0.99 | 1.03 | 1.06 | 0.99 | 1.02 | 1.03 | 1.03 |
| AAU(N) | 0.86 | 0.82 | 0.87 | 0.87 | 0.87 | 0.89 | 0.87 | 0.88 |
| *AAC(N) | 1.14 | 1.18 | 1.13 | 1.13 | 1.13 | 1.11 | 1.13 | 1.12 |
| AAA(K) | 0.99 | 1.00 | 0.99 | 0.97 | 0.98 | 0.99 | 0.99 | 1.00 |
| AAG(K) | 1.01 | 1.00 | 1.01 | 1.03 | 1.02 | 1.01 | 1.01 | 1.00 |
| GAU(D) | 1.16 | 1.16 | 1.15 | 1.14 | 1.15 | 1.12 | 1.15 | 1.13 |
| GAC(D) | 0.84 | 0.84 | 0.85 | 0.86 | 0.85 | 0.88 | 0.85 | 0.87 |
| GAA(E) | 1.06 | 1.05 | 1.06 | 1.08 | 1.05 | 1.06 | 1.06 | 1.06 |
| GAG(E) | 0.94 | 0.95 | 0.94 | 0.92 | 0.95 | 0.94 | 0.94 | 0.94 |
| UGU(C) | 1.10 | 1.14 | 1.15 | 1.11 | 1.13 | 1.14 | 1.12 | 1.14 |
| UGC(C) | 0.90 | 0.86 | 0.85 | 0.89 | 0.87 | 0.86 | 0.88 | 0.86 |
| CGU(R) | 0.06 | 0.06 | 0.05 | 0.08 | 0.05 | 0.05 | 0.05 | 0.05 |
| CGC(R) | 0.19 | 0.17 | 0.19 | 0.19 | 0.19 | 0.19 | 0.22 | 0.22 |
| CGA(R) | 0.19 | 0.22 | 0.19 | 0.29 | 0.19 | 0.19 | 0.22 | 0.19 |
| CGG(R) | 0.28 | 0.31 | 0.30 | 0.32 | 0.30 | 0.27 | 0.30 | 0.30 |
| AGA(R) | **2.72** | **2.75** | **2.71** | **2.68** | **2.75** | **2.71** | **2.73** | **2.70** |
| AGG(R) | **2.56** | **2.50** | **2.55** | **2.44** | **2.51** | **2.58** | **2.48** | **2.54** |
| GGU(G) | 0.80 | 0.81 | 0.79 | 0.79 | 0.85 | 0.83 | 0.81 | 0.81 |
| GGC(G) | 0.73 | 0.71 | 0.73 | 0.74 | 0.70 | 0.73 | 0.72 | 0.71 |
| GGA(G) | 1.01 | 0.99 | 1.02 | 1.07 | 1.03 | 0.98 | 1.02 | 1.05 |
| GGG(G) | 1.46 | 1.49 | 1.45 | 1.41 | 1.42 | 1.46 | 1.45 | 1.44 |

*represents that the different synonymous codon usage pattern between CP biotype (strains 21NX-53, 21NX-300, 21SD-16 and 22NX-9) and NCP biotype (strains 21NM-44, 21NX-69, 21SD-5 and 22NX-197) of the BVDV isolates.

The bold number means the corresponding synonymous codon with the overrepresented usage; the underscore number means the corresponding synonymous codon with the underrepresented usage.
